# The importance of early COPD diagnosis during a smoking cessation program

**DOI:** 10.1186/1617-9625-12-S1-A22

**Published:** 2014-06-06

**Authors:** Eleni Ischaki, Eleni Litsiou, Vasiliki Saltagianni, Ioanna Nikoloutsou, Aikaterini Tsoutsa, Andreas Asimakos, Spyros Zakynthinos, Paraskevi Katsaounou

**Affiliations:** 1 Pulmonary and Critical Care Department, Evangelismos Hospital, Athens, 10376, Greece

## Background

Many patients with mild–moderate COPD (chronic obstructive pulmonary disease), are asymptomatic. Since expressed symptoms are usually mild and mostly attributed to age, they are often underestimated [1,2]. Thus early COPD patients usually remain undiagnosed [3]. The aim of the study is to evaluate the rates of undiagnosed COPD cases in early stages of the disease (stage I and II according to GOLD classification), [4] in our smoking cessation program and to assess the effectiveness of COPD diagnosis as a motivational tool for quitting smoking.

## Materials and methods

551 current smokers, aged ≥18 years old, attended voluntarily the smoking cessation program in our outpatient smoking cessation clinic. All smokers performed spirometry. Behavioral counseling and pharmacotherapy with varenicline were administered to all participants.

## Results

During the study, 85 of 551 smokers were diagnosed for COPD. Only 5 of them were previously diagnosed with the disease (2 in stage II, 2 in stage III and 1 in stage IV). None of them reported symptoms. Smoking abstinence rates at 3 months was recorded. Overall smoking cessation rates three months after behavioral counseling was 55% (n=303). This percentage was higher in first diagnosed COPD patients, as shown in Table 1.

**Table 1:** COPD smokers that attended our Smoking Cessation Clinic

COPD stages	Ι	ΙΙ	ΙΙΙ	IV
**n**	39	35	11	1
**First diagnosis**	39	33	9	0
Abstinence rates after 3 months^*^	30 (76.9%)	30 (85.79%)	7 (63.6%)	1 (100%)

^*^ Abstinence rates are referred to the summation of old and new COPD cases

## Conclusions

A smoking cessation program is a great opportunity to identify undiagnosed COPD cases. COPD diagnosis is an effective motive to quit smoking. Smoking cessation combined with treatment based on COPD severity can modify the progression of the disease. Namely the rate of yearly FEV_1_ decline and COPD exacerbations are reduced after smoking cessation and patients’ health related quality of life is improved. The above effects are maximized when smoking cessation is achieved in early COPD stages [5,6]
